# Co-designing an International Nursing Course on Workplace Violence in Ethiopia, Sweden, and Tanzania: A Collective Autoethnography

**DOI:** 10.1177/23333936251393222

**Published:** 2025-11-14

**Authors:** Martin Salzmann-Erikson, Elisabet Eriksson, Tilahun Saol Tura, Tumbwene Elieza Mwansisya, Loveluck Mwasha, Malin Jordal

**Affiliations:** 1 Department of Caring Science, Faculty of Health and Occupational Studies, University of Gävle, Sweden; 2 School of Nursing, College of Health Science, Wolaita Sodo University, Ethiopia; 3 School of Nursing and Midwifery, The Aga Khan University, Dar es Salaam, Tanzania

**Keywords:** collaborative autoethnography, educator identity, nursing education, workplace violence, transformative learning

## Abstract

This study explores whether and how the co-creation of an international university course on workplace violence served as a site of transformative learning for us as nursing educators across Sweden, Ethiopia, and Tanzania. Using collaborative autoethnography, our team of six faculty members critically reflected on pedagogical assumptions, educator identities, and experiences of cross-cultural collaboration. The data comprised written reflections, meeting transcripts, and observational notes. Through this process, our pedagogical approaches and professional identities were reshaped, shifting from content deliverers to reflective co-learners within the cross-cultural partnership. Our analysis highlights the interplay of cultural negotiation, logistical frictions, and unspoken yet influential power dynamics that shaped the collaboration. These pedagogical transformations extended beyond the course itself, contributing to curricular innovations at our home institutions. By offering thick, reflexive descriptions of educator learning, the study proposes a nuanced model for equitable knowledge co-production, showing that the relational and institutional work of partnership is as critical as the pedagogical content itself.

## Introduction

Workplace violence (WPV) against healthcare workers is a widespread concern with substantial implications for staff well-being, patient outcomes, and healthcare organizations globally. A recent meta-analysis found that, within a 12-month period, approximately 62% of healthcare workers worldwide experienced some form of WPV: 43% non-physical and 24% physical incidents (Liu et al., 2019). A Swedish nationwide survey of 144,000 clinicians found that 34% of physicians and 53% of nurses had encountered workplace incivility in the previous year, with identity-based harassment affecting up to 58% of nurses born outside Europe and co-workers more frequently implicated than patients (Gynning et al., 2025). In Europe, the 12-month prevalence was 48% for any WPV, with 32% reporting non-physical incidents and 20% experiencing physical assault (Liu et al., 2019). The scale of the issue in Africa is significant. Regional and country-specific research highlights how systemic and contextual factors shape both the prevalence and forms of WPV. One meta-analysis of nearly 10,000 nurses found a 12-month WPV prevalence of 62.3%, primarily driven by verbal abuse (51.2%), with physical assaults at 15.1% and regional peaks as high as 79% in Southern Africa (Ekpor et al., 2024). Reinforcing this, another meta-analysis from the continent reported a similar pooled magnitude of 55.64%, highlighting that the risk was significantly higher for women, staff with less work experience, and those working in emergency departments (Eshetie et al., 2025). In Ethiopia specifically, a recent meta-analysis found a pooled WPV prevalence among nurses of 39.4%, with higher rates also observed among women, those early in their careers, and single nurses (Ferede et al., 2025).

Beyond statistics, qualitative research reveals that such widespread WPV, frequently manifesting as subtle bullying, profoundly impacts nurses’ well-being and patient care, often stemming from an organizational culture that requires fundamental change to effectively address these issues (White et al., 2025). Ferede et al. (2025) further situate Ethiopia and similar contexts among the settings most acutely in need of effective and context-sensitive educational interventions. Swedish data mirror these concerns: a focus-group study in intensive care units described violence, often from delirious patients or distressed relatives, as a routine occupational hazard, highlighting the near absence of formal prevention training (Sjöberg et al., 2024).

The consequences of WPV are severe and far-reaching. An umbrella review of meta-analyses found that WPV is associated with significant physical, psychological, and occupational harm to healthcare workers (Sahebi et al., 2022). The impact extends beyond immediate harm: Berger et al. (2024) demonstrated that WPV exposure is linked to higher burnout, anxiety, and turnover intention among staff, while O’Brien et al. (2024) highlighted associations with increased medical errors and compromised patient safety. Despite the global prevalence and impact of WPV, recent reviews (Solorzano Martinez & De Oliveira, 2021; Somani et al., 2021) reveal that interventions for nursing students and nurses addressing WPV vary in format.

These interventions vary in format, from standalone workshops to multicomponent programs, and while approaches such as simulation, role-play, and cognitive rehearsal can improve confidence and skill, single interventions often yield only limited reductions in actual WPV rates. Critically, there remains a scarcity of contextually adapted curricula and, where resource constraints and unique local risk factors may shape both the experience of violence and the feasibility of prevention. Developing curricula that aid students to develop cultural competence and a broader worldview may foster capacity-building through exchange of knowledge, and strengthen the nursing profession on global issues, such as WPV. Although research about internationally driven courses is little, Chiuzi (2023) reported that intentionally engaging students, industry representatives, and faculty in a “tri-party” structure leads to greater learner satisfaction and perceived skill acquisition, particularly when interactive, context-specific case discussions are co-developed. Chao et al. (2010) similarly found that online courses achieved higher quality standards when faculty and instructional designers engaged in iterative dialogue around learning objectives, assessment strategies, and student experience, instead of relying on a single author or template.

Yet, despite these recognized advantages and the widespread call for better WPV prevention education, there remains a notable gap in nursing education research: few studies have examined, in reflexive detail, how nurse educators from different health systems collaborate to co-create WPV curricula. While some institutions have developed local modules, little is known about the real-world dynamics of cultural negotiation, logistical barriers, and pedagogical debates that emerge when faculty from different countries pool their expertise. In practice, WPV content is often uneven or siloed, and courses tend to be designed unilaterally, even though nurses in diverse settings share common vulnerabilities to violence. Joint development processes promise more robust and culturally attuned curricula, but empirical accounts of these processes remain rare. The need for such collaborative approaches is particularly pressing which was the origin of our collaboration, a joint effort between Ethiopia, Sweden, and Tanzania. Also, recent evidence highlights the problems nurses face with WPV in our countries (Ekpor et al., 2024; Ferede et al., 2025; Sjöberg et al., 2024; Solorzano Martinez & De Oliveira, 2021). Faculty in these regions face distinct challenges related to resources, technology, and context-specific risk factors. This underscores the need for international curriculum development that responds to local realities and draws on diverse perspectives.

While local initiatives to address WPV are valuable (Amara et al., 2024), the nature of violence itself demands a collaborative international approach since the issue is deeply embedded in cultural, institutional, and power-laden contexts. Developing WPV curricula in isolation risks imposing a narrow, culturally bound definition of violence, often reflecting perspectives from high-income countries where most interventions are designed (De Raeve et al., 2023; Sochan, 2008). In contrast, scholarship on the internationalization of nursing curricula argues that cross-cultural partnerships are essential for developing a pedagogy that is globally relevant (Davey, 2023; Galan-Lominchar et al., 2024). As Haug and Jacobs (2023) contend, the goal of such collaborations is not merely to compare different contexts, but to engage with pluriform perspectives to enable the co-creation of new, shared understandings. The tangible benefits of this approach are empirically supported; Hua et al. (2023), for example, demonstrated that international online courses significantly enhanced nursing students’ intercultural sensitivity, a core competency for professional practice. Furthermore, the very process of collaborative development is critical for achieving an equitable outcome. In any Global North-South partnership, particularly one funded from the North, there is an inherent risk of reproducing a neocolonial view where the Global North partner implicitly sets the agenda (Sochan, 2008). A dialogic and co-creative process, as advocated by Chao et al. (2010), is therefore not just a pedagogical preference but an ethical necessity to mitigate these power imbalances. It is through this shared, often challenging, relational work that a truly robust curriculum can be built. However, empirical research documenting how such cross-national partnerships unfold in practice, particularly in the design of WPV curricula for nursing, is, to our knowledge, non-existent.

### Aim

The aim of this study is to explore whether and how educators’ personal and collaborative reflections shaped the design, implementation, and pedagogical evolution of an international course on workplace violence in healthcare.

## Methods and Materials

Our inquiry was grounded in a post-qualitative orientation, which assumes knowledge to be relational, situated, and continuously in process (St. Pierre, 2021a, 2021b). This orientation resists the idea of method as a fixed set of procedures and instead emphasizes knowledge production as emergent and relational.

Autoethnography bridges autobiographical narrative and sociocultural analysis, enabling subjectivity to be used as an analytic resource rather than treated as bias (Ellis et al., 2011; Spry, 2001). In its collective form, it allows multiple voices to be interwoven dialogically across cultural and institutional contexts, thereby capturing relational dynamics that are otherwise difficult to grasp (Butz & Besio, 2009). Collective autoethnography has also been recognized as particularly powerful for making visible the epistemic and affective dimensions of collaborative academic work (Stahlke Wall, 2016). In nursing education, autoethnographic approaches have been used to explore educators’ and students’ lived experiences with identity, bias, and pedagogy, providing a nuanced understanding of the cultural and ethical dimensions of educational practice (Douglas & Carless, 2025; Mayoum et al., 2022; Salzmann-Erikson, 2024a).

Within this stance, we employed collective autoethnography as a methodological practice. This approach was particularly suited to our study because the process of co-developing an international course is inherently reflective, relational, and shaped by transcultural collaboration. At the same time, our focus on workplace violence in nursing education required a methodology capable of accommodating power asymmetries, institutional differences, and culturally situated perspectives on pedagogy. By engaging in systematic self-reflection and dialogue, our team of educators from Ethiopia, Sweden, and Tanzania analyzed how diverse values and experiences influenced the design and delivery of the course. Thus, collective autoethnography did not merely serve as a representational tool but as a practice that materialized our post-qualitative stance, allowing inquiry to unfold through the interplay of subjective experience, cultural diversity, and theoretical reflection.

### Study Context

The research focuses on the collaborative development and implementation of an international course addressing workplace violence in healthcare. The course was created as part of a larger Erasmus+ International Credit Mobility project (running the period 2023–2026) involving teacher and student mobility in higher learning institutions from three different countries: Ethiopia, Sweden, and Tanzania. While funding was secured through the Swedish Higher Education Authority and the project was coordinated by University of Gävle, the course was formally based at University of Gävle in several key respects: all students, regardless of home institution, were registered through Gävle’s digital admission system; the course syllabus and credits (4.5 ECTS) were anchored in Gävle’s academic regulations; teaching and examination were conducted using Gävle’s digital learning platform (Canvas); and final grading and transcript documentation were issued by University of Gävle. Although the collaboration was initiated by the project leader (M.J.), course content and teaching activities were jointly developed and delivered by educators from all three partner countries. The course “Workplace Violence in Healthcare” thus became a shared platform for co-creation, fostering mutual understanding and shared learning among participants from diverse backgrounds. This autoethnography captures the reflective experiences of educators as we navigated challenges and opportunities inherent in this collaborative, cross-cultural educational endeavor.

### Participants

Six educators, who also serve as the co-authors of this study, participated in the reflective, autoethnographic process of co-designing the course. The broader course team included one additional Ethiopian colleague who contributed to course delivery but did not take part in the research component and is therefore listed in the Acknowledgment. The six co-authors comprised three from Sweden (the course examiner [M.S.-E.] and two course coordinators [E.E. and M.J.]), two from Tanzania (T.S. and L.M.), and one from Ethiopia (T.M.). Although we held differing formal titles (examiner, coordinators, and instructors) all six co-authors contributed to curriculum design, teaching, and reflection.

### Data Collection

Data were collected throughout the planning, implementation, and evaluation of the international course and comprised three complementary categories: (1) individually written reflections by the authors, (2) transcripts from selected online meetings, and (3) email correspondence across the trinational team. We held brief “reflection check-ins” immediately after each major planning meeting (approximately bi-monthly during the 9-month design phase), following key seminars, and after the course concluded. All co-authors contributed to the reflective material by documenting personal experiences, tensions, and insights in relation to course development and teaching. These reflections, some contemporaneous and others retrospective, were written individually or shared informally in team communication and later curated for analysis. The length and style varied from shorter notes to extended narratives, and not all experiences were documented in writing; much of our collective sense-making occurred orally in real-time meetings, highlighting the limits of relying solely on written traces to capture collaboration (Cole & Scribner, 1981; Ong, 2012). In addition, we drew on a series of thirteen online meetings held between January 2023 and August 2025. These sessions varied in duration from 30 to 90 min, with a median length of around 1 hr. While only a selection of the meetings was transcribed in full, they included key points in the process such as course plan development, admission procedures, scheduling, and post-course debriefs. Taken together, these materials provided a longitudinal record of evolving dynamics and decisions. Email exchanges among the Ethiopian, Swedish, and Tanzanian colleagues were also included, providing insight into administrative processes, expectations around deadlines, and moments of miscommunication or negotiation. Taken together, these three types of material constitute a multimodal dataset that reflects both the immediate realities of working across institutional, cultural, and digital boundaries and the evolving interpretations and meaning-making that occurred through individual and collective reflection. No structured interviews were conducted; instead, the organic flow of collaboration served as the field of inquiry, with written and spoken traces forming the empirical foundation for this collective autoethnographic analysis.

### Data Analysis

Our analytic process was situated within a post-qualitative inquiry, which resists the reduction of data into categories through conventional coding (St. Pierre, 2021a; St. Pierre & Jackson, 2014). Instead, analysis was enacted through collective autoethnography as a relational and reflexive practice (Butz & Besio, 2009; Chang, 2008). The material was read carefully, and words or phrases that stood out were noted, not as systematic codes but as traces carrying affective or conceptual weight. These traces were introduced into group dialogues, where they were examined across cultural and institutional perspectives. Rather than seeking consensus, we treated divergent interpretations and contradictions as analytically useful, reflecting our commitment to polyvocality (Burleigh & Burm, 2022; Ellis et al., 2011; Salzmann-Erikson, 2024a).

Through iterative cycles of reflective writing, discussion, and rewriting, broader orientations gradually took shape. These orientations included collaboration norms, logistical challenges, dynamics of power, pedagogical transformation, and emotional dimensions. Over time, they shifted and evolved throughout the process. For instance, an initial emphasis on “project leadership and power dynamics” later transformed into “unspoken power dynamics,” illustrating how labels and meanings were renegotiated over time. In this way, analysis became less about classification and more about inhabiting the movements of thought, affect, and relation within our collaboration. Rigor, in this context, was not grounded in procedural systematics but in reflexivity, transparency, and the preservation of multiplicity (Salzmann-Erikson, 2024b; St. Pierre, 2021b).

### Ethical Considerations

All participants gave informed consent to share their reflective narratives as part of this study. Because the material contained no patient data or sensitive personal information, only co-authors’ professional reflections on their own teaching practice, the project did not require institutional ethical review (SFS, 1993). Nevertheless, the principles articulated in the Declaration of Helsinki (WHO, 2013) guided our work, particularly those concerning respect for persons, integrity, and transparency in research.

Still, ethical issues in autoethnography cannot be reduced to procedural compliance. Lapadat (2017), in her article specifically addressing ethics in autoethnography and collaborative autoethnography, emphasizes that such inquiries are inherently ethical because researchers represent themselves rather than appropriating others’ voices. At the same time, they raise relational risks by exposing vulnerabilities and potentially straining professional relationships. In our case, when we discussed our differences, we exposed ourselves to discomfort and the possibility of conflict. Yet we found that this openness fostered deeper collaboration: by voicing tensions honestly, we strengthened mutual trust and relational bonds. This reflects what Lapadat (2017) terms *relational ethics*, an iterative practice of balancing honesty and care in collaborative research. We collectively navigated these challenges by revisiting what to disclose, how to represent tensions, and how to ensure fairness in voice representation. Ethics was thus not a static requirement, but a living practice enacted throughout the project, integrating procedural safeguards with ongoing reflexive accountability.

## Results

The findings from our collective autoethnographic reflections are presented in three interconnected themes. These themes emerged through collaborative analysis of meeting transcripts, written reflections, and in-person exchanges, where individual narratives were interwoven into shared patterns. Each theme represents a synthesis of our individual narratives, discussed and reframed collectively until shared patterns became visible. Together, they illuminate how the course was designed, implemented, and pedagogically evolved. The first theme, *Digital and Logistical Frictions*, captures how uneven digital conditions and practical constraints shaped the everyday work of course design and delivery. The second theme, *Negotiating Cultural and Institutional Differences*, highlights how varying academic roles, expectations, and communication practices influenced decision-making, power dynamics, and collegial trust. The third theme, *Pedagogical Transformation through Co-Design*, shows how the collaborative development of the course reshaped our understanding of workplace violence and shifted our educator identities toward more reflexive, student-centered practices. By presenting these themes, we demonstrate how cross-national collaboration not only involved navigating technical and institutional frictions but also fostered deeper professional and pedagogical transformation.

### Digital and Logistical Frictions

This theme shows how unstable internet, platform differences, and travel logistics shaped daily collaboration. These frictions disrupted meetings but also influenced how we adapted course design and implementation. The practical implementation of the course design relied heavily on digital meetings on Zoom, which were common throughout the initiation, development, and execution of the course, yet these sessions often felt stilted; restricted to brief agendas, hampered by muted microphones, and interrupted by frequent dropouts. From the outset, it was clear that we were navigating a wide spectrum of digital conditions. Scheduled calls were sometimes delayed or missed because of unstable internet connections; as one colleague remarked during a reflection meeting, “Last time they had some common, you know, internet problems,” and later, “He’s in X-town. . . maybe internet problems.” (A reflection made by one of the Ethiopian colleagues).

Participants in Sweden found such interruptions unfamiliar and frustrating. This was unsurprising since university offices and homes typically have 10–100 Mbit/s fiber connections and widespread 5G. By contrast, colleagues joining from Tanzanian and Ethiopian campus offices contended with unstable bandwidth or struggled to find quiet spaces for private discussions. During several sessions, a participant began in one room only to be asked to relocate by a colleague who needed the space. Video-conference etiquette and varying equipment added further complexity. As a Swedish colleagues reflected, “Several participants had malfunctioning cameras, and some never turned theirs on,” adding that “some left their microphones on even when it was not their turn to speak.” These lapses repeatedly interrupted discussions of the course content. Occasionally, the virtual classroom echoed with unexpected soundscapes: “It was constantly disrupted by background noise—chickens clucking, people speaking in the same room as participants.” That chorus of rural life reminded us that our course spanned environments ranging from silent lecture halls to bustling home compounds, each with its own soundtrack.

Each disruption required the team to renegotiate time slots, resend links, or switch from live to asynchronous formats. To bridge this distance, we organized a mobility teacher exchange just before and during the course launch: two educators from each partner university travelled together and spent two intensive weeks on the Swedish campus as part of a mobility program. Sharing offices, commuting on the same local buses, and queuing side-by-side in the staff cafeteria offered a slow-burn immersion that no Zoom call could replicate. Free from pixelated windows and time-pressured agendas, we lingered over rubrics, observed one another’s facial expressions, and read posture as closely as prose. Shared laughter punctuated these sessions, dissolving formalities and nurturing a sense of camaraderie. This collective joy built trust and sustained our later online collaboration. These face-to-face sessions transformed our planning from transactional check-ins into deep, generative dialogue; allowing us refine grading criteria, and craft truly inclusive pedagogical strategies with a nuance virtual rooms rarely permit.

The journey itself, however, surfaced fresh logistical challenges. Upon arrival in Sweden, members of the Tanzanian and Ethiopian teams discovered that the cash they had brought could not be used to purchase train tickets to their final destination: cash transactions were not accepted in most Swedish settings, where an estimated 92 % of all payments are electronic. One colleague reflected, “I had the cash, but I couldn’t do the transaction. . . I started roaming around the airport trying to find how I could commute.” A society in which cash is useless was unheard of in an African setting, just as travelling without a debit or credit card was difficult for Swedish colleagues to imagine.

By facing these logistical barriers head-on, we turned them into collective learning opportunities. Errant microphones and network drop-outs became prompts for empathy; Canvas (a student learning platform) mix-ups reminded us that no single platform can serve all contexts perfectly. Subsequent online meetings were markedly less frustrating, as we could now “see beyond” cracked voices, poor video quality, and background noise.

### Negotiating Cultural and Institutional Differences

This theme highlights how contrasts in academic roles, communication, and hierarchy required negotiation and adaptation, shaping collegial trust and decision-making. From the moment we embarked on this tri-national collaboration, questions of authority, responsibility, and curricular scope surfaced alongside our pedagogical goals, fundamentally shaping the course’s eventual design and implementation. Although the course was institutionally anchored at the University of Gävle, roles and decision-making authority shifted dynamically among our diverse team. Early on, a colleague from Sweden learned he would serve as course examiner, a responsibility he had not anticipated: “I had not thought of the process through the examiner’s lens, but once appointed I realized I must be meticulous so that nothing backfires when the course launches.”

This surprise appointment highlighted how formal Swedish academic roles carry precise obligations, whereas collegial teamwork elsewhere is often less legally codified. One of the Swedish colleagues, as the grant recipient, held ultimate administrative stewardship. Another served as course examiner, a role conferred by the department head that carried authority over assessment and was distinct from the broader project leadership. The concentration of both high-stakes roles within the Swedish team thus risked reproducing inequality in our tri-national collaboration, even as we strove for reciprocal partnership.

Respecting these differences while maintaining course functionality became central to our negotiations. Balancing responsibility with differing work rhythms required its own exercise in power-sharing. We introduced clear response protocols to reduce ambiguity. These included follow-up emails after each meeting and explicit deadline confirmations. Rather than issuing rigid ultimatums, we framed reminders as supportive check-ins and remained open to extensions when needed. These adjustments upheld both the integrity of our pedagogical design and the trust underpinning this international endeavor.

Even when commitments were met across the partnership, contrasting norms around contesting instructions or negotiating deadlines sometimes led to last-minute uploads and unspoken tensions. Conversations about “African” and “Swedish” realities, though reductive, helped illuminate shared experiences among Ethiopian and Tanzanian colleagues, which often contrasted with Swedish perspectives. During a meeting, a Swedish colleague paused the discussion to point out that, when referring to the partnership, we sometimes slipped into continental binaries: “It’s maybe to bring up that it’s very easy to fall into those two bigger groups—Africa and Sweden, Africa, Sweden [. . .] it’s broad categorizations that are not without so much nuance.” A Tanzanian team-mate echoed the concern:

“Very true [. . .] had it been *Ethiopia and Tanzania*, I think we would have noted a lot—a lot—of differences among the group.” These reflections reminded the team that a single “African” perspective does not exist; instead, multiple national, institutional, and personal lenses shape how WPV is understood and taught.

The institutional differences were also reminded of when we discussed pedagogy and administrative tasks. A colleague voiced different expectations about what needed discussion or elaboration, requiring extra energy to articulate their priorities. As a Tanzanian colleague explained:

*And sometimes you are in a meeting and it doesn’t make sense at all. And then you ask yourself, okay, this is what they are saying. But is it necessary? Or why don’t we put this? Because to me it is important. To me it is an issue.* [. . .] *you could see that some of the things that we feel that they are an issue, they are not necessarily. And it takes that extra effort to try to make your point so that your colleagues can realize, okay, yeah, this makes sense. You have to use that extra energy to explain something like that.*

Physical meetings exposed further cultural assumptions. Ethiopian and Tanzanian colleagues arriving in Sweden found the absence of a welcoming party at the airport, 140 km from the university, as disorienting. While independent travel is normal in Swedish academic culture, East-African customs value direct hospitality. One colleague noted, “Back home, a ride from a familiar face is a given.” The logistical gap—exacerbated when electronic payments failed—highlighted how norms around arrival and support are culturally embedded.

Another issue concerned expectations around meetings, deadlines, and email responses. The course leader observed, “I often receive very little response to my emails, and I’m unsure whether everyone has seen the deadline or opted out.” She wondered whether silence indicated reluctance, uncertainty, or a different communication style. Reflecting later, a Tanzanian member remarked that Swedish partners were equally cautious not to criticize: “You don’t want to criticize us, but we don’t want to criticize you.”

These unspoken silences revealed how power, culture, and infrastructure shape operational flow and shared agency. Because the collaboration depended on funding and Swedish regulations, pressing too hard risked alienating partners or reinforcing Global North–South hierarchies. By openly acknowledging these dynamics, we modelled culturally attuned leadership that can transform structural constraints into collaborative strength.

### Pedagogical Transformation Through Co-design

This theme shows how co-designing the course reshaped our understanding of workplace violence and shifted our roles from instructors to co-learners, encouraging more reflexive and student-centered practice. The process of co-designing the course was not merely a technical task of curriculum development; it became the engine of a shared pedagogical evolution that repeatedly turned our analytic gaze inward. Early in our collaboration, colleagues from Ethiopia and Tanzania spoke of workplace violence almost exclusively in physical terms, such as assaults, threats, or visible aggression. In contrast, we as Swedish facilitators had assumed a broader spectrum that included verbal harassment and institutional neglect. Through seminar dialogues and co-teaching exercises, these narrow definitions were challenged: peers contributed examples of emotional abuse, micro-aggressions, and systemic intimidation. By the close of our first module, the team had co-authored a shared framework recognizing WPV as a multifaceted phenomenon. This conceptual shift underscores how our collective autoethnographic process reshaped not only our strategies but also the very language we used to talk about violence. That reframing immediately fed back into our seminar design, prompting us to rethink how we engaged learners. We began with familiar lecture-driven routines, yet our first Zoom debriefs made it clear that this format risked positioning students as passive recipients. In response, we re-oriented every seminar toward *reflexive praxis*: prompts invited learners to connect WPV theories to their own workplaces, and we practiced “listening first,” allowing productive silence to do its work.

This pedagogical re-set also altered our identities. We deliberately adopted *co-learner* roles, sharing personal narratives of clinical violence and acknowledging where we were still learning. Colleagues described how the course had affected their students’ understanding of workplace violence and how it had reshaped their own thinking and awareness of workplace violence in their respective healthcare settings. During a reflection meeting a Tanzanian educator explained:

*Some of them* [author’s note: students] *. . . were quite excited; they felt they now had a reason to stand up and argue instead of keeping silent. They can see the oppression, but no one is ready to speak out. Some have already organised sessions at work, talking about how a ‘harmless’ comment can hurt a colleague.*

Additionally, colleagues began to reflect on their own participation in oppressive structures that became visible through the transformative work of designing and teaching the course. Later, the same colleague added:

*For me, the process has made me scrutinize my day-to-day interactions—sometimes we oppress unconsciously because of our position. When I teach master’s students, many of whom are managers, I ask them to share how they have felt oppressed and how they may have been oppressors. I am one of those who have been transformed.*

These extended reflections show how educator and student learning intertwined: modelling vulnerability encouraged a shift from silence to voice, from individual grievance to collective critique. A Swedish colleague later emailed, “The classroom feels more *student-owned* than anything I’ve facilitated before.” These reflections made clear how we began noticing our own everyday assumptions and behaviors. By sharing our vulnerabilities, we shifted from silently enforcing hierarchies to actively inviting students, and we found ourselves reframing the teacher role as co-learners alongside our students.

The redesign also redistributed responsibility. Together we drafted guiding questions that asked students to interrogate institutional policies and propose context-sensitive mitigation strategies; facilitation rotated among Ethiopian, Tanzanian, and Swedish staff, signaling that no single geography held epistemic primacy. We redesigned assessment to fit both group interaction and individual reflection. First, students met in small groups to discuss an assigned article, prepare a 2–4-slide PPT summarizing key concepts and personal insights, and present it in a 20-minute seminar. Then each student wrote an individual essay with the prescribed headings: Introduction, Empirical Experience, Analysis, Strategies. The essays wove together a personal or hypothetical case with course literature and followed the university’s formatting and citation rules. This two-stage format kept everyone engaged, distributed workload evenly, and worked smoothly across different internet speeds and grading systems. In this way, the course became a living laboratory for transformative learning: curricula, instructors, and students evolved together, demonstrating that cross-cultural reflexivity can unsettle entrenched hierarchies and nurture professional agency on all sides.

## Discussion

This study critically examined how co-creating an international course on workplace violence against healthcare workers transformed our pedagogical perspectives and practices as faculty members across Ethiopia, Sweden, and Tanzania. The three themes presented in the results directly reflect our stated aim: the first addresses course design, the second implementation, and the third the pedagogical evolution of our teaching roles. In the discussion that follows, we integrate these findings with prior scholarship in nursing education, critical pedagogy, and global academic collaboration.

Our differing contexts initially shaped how we understood and approached workplace violence, but co-developing the course made these contrasts visible and generative. We began this collaboration with the aim to build a course that was context-sensitive and reciprocal, where all partners could learn from each other and from students. Our findings indicate that co-developing the course was a transformative learning experience for the educators involved (Mezirow, 1991; Taylor & Cranton, 2012). We each entered the project with our own teaching paradigms and assumptions, yet the cross-context collaboration served as a disorienting dilemma that challenged those assumptions and prompted critical reflection. One striking example was how our shared understanding of workplace violence itself was reshaped: several colleagues from Ethiopia and Tanzania initially understood WPV as predominantly physical, whereas the course discussions and co-teaching activities surfaced new layers of meaning. Through dialogic exchange, psychological and non-verbal forms of violence, such as exclusion, intimidation, or institutional neglect, gained visibility. All partners experienced a shift in understanding, though they began from different cultural and professional horizons. These mutual “a-ha” moments show how context and culture shape even basic concepts, enriching both the course content and our collaborative practice. This responds to calls in the literature for more contextually grounded WPV curricula in low- and middle-income countries (Solorzano Martinez & De Oliveira, 2021), addressing the limitations of existing interventions developed in high-income contexts (Ekpor et al., 2024; Liu et al., 2019). These shifts catalyzed changes in how we understood ourselves as educators and how we approached curriculum and instruction. Addressing asymmetrical power and yearning for more equitable collaborations and the recognition of diverse epistemologies is described to be of importance in international collaborations (Anjum & Aziz, 2024; Choquez-Millan et al., 2024). Applied to curriculum design, these principles mean that collaboration cannot be restricted to just an exchange of content but requires mutual recognition and shared participation: we had to actively validate and incorporate the pedagogical approaches and experiences of each partner institution.

In light of Mezirow’s theory of transformative learning, our experiences can be seen as perspective transformations encompassing psychological, convictional, and behavioral dimensions (Mezirow, 1991; Taylor & Cranton, 2012). Psychologically, we experienced changes in our understanding of ourselves as teachers. In terms of pedagogical convictions, several of our long-held beliefs were revised after seeing alternative approaches succeed in different cultural contexts. Behaviorally, we altered our teaching practices, for example by incorporating more participatory, case-based learning activities. These shifts align with the notion that transformative learning revises worldview and self-perception, often through cumulative critical reflections. These shifts align with the notion that transformative learning revises worldview and self-perception, often thought cumulative critical reflections. As educators, we not only revised our pedagogical convictions but redefined our professional identities. The process of reflecting together through personal narratives and dialogic feedback fostered a shift from content deliverers to co-inquirers. These shared learnings also shaped how we collectively redefined WPV, not as a fixed category but as a layered context-sensitive phenomenon. This was reported in our results, particularly in how course co-design reshaped our definitions of violence, our teaching practices, and our understanding of cross-cultural partnership.

Notably, some colleagues also reported that their engagement in this course development had begun to influence their revised teaching practices beyond the immediate scope of this project. Some colleagues’ transformation was particularly evident in how they began to scrutinize their pedagogical stance. One educator, for example, started reflecting critically on how her role as a teacher might unintentionally reinforce silence or hierarchy when addressing workplace violence. Such moments exemplify the kind of critical self-reflection that lies at the heart of transformative learning (Mezirow, 1991), where teaching becomes not only a delivery of content, but a situated, ethical practice shaped by power and positionality. This type of leap has also been documented in other cross-cultural educational collaborations, where participation in global health or development-focused teaching initiatives led to sustained pedagogical innovation at the institutional level (Hennessy et al., 2015; Hökkä et al., 2017; Sowton & Thornbury, 2021; Zou et al., 2024).

This shift from deliverers of content to co-learners and inquirers of pedagogy marked a profound change in subjectivity. It reshaped how we understood and positioned ourselves as educators. Rather than viewing ourselves as authoritative transmitters of knowledge, we began to experience our roles as dynamic, relational, and shaped by mutual vulnerability and learning. It also had a humanizing effect: we came to see each other (and ourselves) not just as representatives of institutions or countries, but as individuals growing through vulnerability and reflection. This reflective process extended beyond the course itself. Writing this article prompted us to articulate shared difficulties and deepen our mutual understanding. Co-authorship thus became a continuation of our pedagogical transformation, enabling us to revisit moments of uncertainty and reframe them as collective learning. This transformation mirrors findings from studies on teacher educators’ professional development, where sustained engagement and the sharing of personal and professional narratives have been shown to foster a stronger collective identity and agency. Such processes, built on mutual trust and recognition, create an atmosphere where educators move beyond formal roles to connect as authentic individuals, enabling deeper reflection and collaborative growth (Hökkä et al., 2017).

Yet, our commitment to egalitarian dialogue did not fully erase underlying power dynamics. Unspoken hierarchies rooted in global academic structures and cultural norms still lurked in the background, previously discussed in postcolonial critiques of North–South academic collaborations (Anjum & Aziz, 2024; Choquez-Millan et al., 2024). The partners from the high-income country (Sweden) were inevitably perceived as the donor and conceptualizer to some degree, while the partners from Ethiopia and Tanzania risked being seen as local implementers of teaching. A possible explanation for this perception may lie in the structural origin of the project: it was the Swedish university that had formally secured the funding and thus held the administrative and financial coordination responsibilities from the outset. This aligns with recent studies showing that structural funding arrangements often reproduce epistemic inequalities in international partnerships (Anjum & Aziz, 2024; Choquez-Millan et al., 2024). Yet, it should not be underestimated that the persisting North-South inequalities entrenched in institutions and human psyches contribute to unconscious and taken-for-granted power imbalances. This is something to be mindful of in collaborations between high- and low-income countries.

Cultural norms of collaboration and communication shaped these power dynamics. Unspoken rules such as deference to seniority or reluctance to disagree varied across cultures and could silence some voices if left unaddressed. Upon prompting for what largely had remained “unspoken” during the course development, it surfaced that the Swedish colleagues refrained from voicing criticism towards the African participants, which could be a sign of fearing to reinforce historical and global inequalities. However, such fear of claiming responsibility may instead reinforce unequal power hierarchies.

Yet, we conclude the rich synergy that we achieved through our collaboration was predicated on our willingness to engage in dialogue across differences, which is a hallmark of both critical pedagogy and successful international partnerships. Our autoethnographic approach gave us a forum to reflect on these culturally inflected power dynamics honestly. Writing and exchanging our personal narratives unveiled how each of us perceived the power balance, enabling collective adjustments.

### Methodological Considerations

Our use of collaborative autoethnography, situated within a post-qualitative, enabled us to generate knowledge within the team through dialogic reflection. (Butz & Besio, 2009; St. Pierre, 2021b). Thick descriptions of lived pedagogical experiences, we sought to convey not only what happened but how it was subjectively and affectively experienced, to preserve contextual nuance and embodied meaning (Denzin, 2014; Tardy, 2021). Still, questions of credibility and rigor must be addressed. To enhance trustworthiness, we engaged in reflections, triangulated interpretations across cultural perspectives, and maintained reflexive transparency in how we curated narratives. However, rigor was not grounded in coding or procedural systematics but in reflexivity and the preservation of multiple voices (Ellis et al., 2011; Salzmann-Erikson, 2024b). Voice representation was a key concern: rather than smoothing differences, we allowed divergent views to remain visible. By including multiple voices, sometimes diverging, we sought to enact polyvocality not only as an analytic principle but also as a way of writing.

Our dual role as both participants and researchers presents both a methodological strength and a potential limitation. The primary strength lies in the rich, insider perspective this affords, allowing for a nuanced analysis grounded in lived experience that would be unavailable to an external observer. The process of co-writing itself became an extension of our analysis, enabling a dialogic deepening of our reflections. However, this very embeddedness creates limitations. As co-authors invested in maintaining positive professional relationships, there was a shared reluctance to voice direct criticism, a dynamic captured in one reflection: “You don’t want to criticize us, but we don’t want to criticize you.” This could risk smoothing over analytically important tensions. We addressed this challenge by embracing our autoethnographic stance. Instead of forcing consensus when interpretations differed, we used the collaborative writing process as a forum to make these very tensions visible and treat them as productive. For instance, during our analysis, we noted a tendency to frame our collaboration as a binary between “Africa” and “Sweden.” Rather than resolving this, we engaged in a dialogue that highlighted the internal diversity within that binary. By preserving polyvocality, we turned the potential limitation of our dual roles into a source of reflexive strength, allowing the analysis to remain complex and multi-layered.

Nonetheless, some limitations warrant acknowledgment. As participants and researchers, we were inevitably entangled in the process of meaning-making, which introduces risks of selective memory and overemphasis. Our deep engagement in the process may also have led to potential blind spots regarding the influence of institutional and structural constraints. Similarly, while we acknowledge that sex and gender are critical dimensions of WPV, these factors did not emerge as a central theme in our collective analysis of the *collaborative process* itself, and a deeper exploration of them was therefore beyond the scope of this particular study. While thick descriptions enhance contextual credibility, the absence of external validation (e.g., student feedback or observational data) limits the generalizability of our claims. Furthermore, the high level of trust and openness required for collaborative autoethnography may not be replicable in all teams or institutional environments (Adams & Herrmann, 2023; Ellis et al., 2011).

### Implications for International Educational Partnerships

Our study offers important insights into educational practice. A key implication is that the process of co-design itself should be viewed as a vital form of professional development. We found that our most significant learning emerged directly from navigating the challenges of the collaboration; the frictions of differing pedagogical views, communication styles, and unspoken power dynamics. This suggests that for similar partnerships to succeed, the relational work of building trust and mutual understanding is not a preliminary step, but rather a core component of the pedagogical project itself. Therefore, institutions should ensure that such projects have dedicated time and resources specifically for this ongoing, reflective dialogue.

Furthermore, our experience highlights a critical institutional challenge: the friction between the administrative procedures of the coordinating institution and the collaborative needs of the partnership. We learned that the bureaucratic requirements of the Global North partner regarding deadlines, student admission, and formal documentation are not neutral. They carry culturally specific assumptions that, if applied without negotiation, can create barriers and unintentionally reinforce the very hierarchies the partnership seeks to overcome. A central implication, especially for coordinating institutions in the Global North, is the need to critically examine their own administrative practices. Project leaders have a responsibility to not only understand these internal systems but to also actively translate and adapt them in dialogue with partners. This work is essential for ensuring that institutional logistics support, rather than undermine, the partnership’s goals of reciprocity and shared pedagogical ownership.

## Conclusion

Co-creating this international course was more than a project in curriculum design; it was a form of critical professional development that transformed us while learning opportunities for students. Our pedagogical transformation was evidenced by a shift towards more participatory, context-sensitive teaching practices and a commitment to continual critical reflection. Educator identities evolved in tandem with new insights into power, dialogue, and knowledge co-production. These transformations echo and extend recent literature in nursing and health professions education, offering a model for future international partnerships grounded in reciprocity, critical consciousness, and pedagogical innovation.
